# West Nile Virus Genetic Diversity is Maintained during Transmission by *Culex pipiens quinquefasciatus* Mosquitoes

**DOI:** 10.1371/journal.pone.0024466

**Published:** 2011-09-12

**Authors:** Doug E. Brackney, Kendra N. Pesko, Ivy K. Brown, Eleanor R. Deardorff, Jon Kawatachi, Gregory D. Ebel

**Affiliations:** Department of Pathology, University of New Mexico School of Medicine, Albuquerque, New Mexico, United States of America; University of Texas Medical Branch, United States of America

## Abstract

Due to error-prone replication, RNA viruses exist within hosts as a heterogeneous population of non-identical, but related viral variants. These populations may undergo bottlenecks during transmission that stochastically reduce variability leading to fitness declines. Such bottlenecks have been documented for several single-host RNA viruses, but their role in the population biology of obligate two-host viruses such as arthropod-borne viruses (arboviruses) *in vivo* is unclear, but of central importance in understanding arbovirus persistence and emergence. Therefore, we tracked the composition of West Nile virus (WNV; *Flaviviridae*, *Flavivirus*) populations during infection of the vector mosquito, *Culex pipiens quinquefasciatus* to determine whether WNV populations undergo bottlenecks during transmission by this host. Quantitative, qualitative and phylogenetic analyses of WNV sequences in mosquito midguts, hemolymph and saliva failed to document reductions in genetic diversity during mosquito infection. Further, migration analysis of individual viral variants revealed that while there was some evidence of compartmentalization, anatomical barriers do not impose genetic bottlenecks on WNV populations. Together, these data suggest that the complexity of WNV populations are not significantly diminished during the extrinsic incubation period of mosquitoes.

## Introduction

West Nile virus (WNV; *Flaviviridae*, *Flavivirus*) was introduced into North America in 1999 and has since spread across the continental United States and into Canada, Mexico, the Carribean, and South America [1]. Molecular epidemiologic studies of WNV in the US revealed that minor changes at the genomic level were associated with a dramatic shift in the genotypic composition of WNV circulating in North America [2]–[6]. Specifically, the introduced genotype, termed NY99, was displaced by a new variant, WN02. The WN02 genotype differs from NY99 by only a few nucleotide and/or amino acid changes, but is more efficiently transmitted by native *Culex* mosquitoes [5], [7], [8]. It was determined that the WN02 genotype requires a shorter extrinsic incubation period in mosquitoes (EIP, time from vector infection to transmission) thereby resulting in an increased vectorial capacity of local mosquitoes. Similarly, the emergence of Chikungunya virus (CHIKV; *Togaviridae*, *Alphavirus*) seems to have been facilitated by analogous mutations that result in increased transmission efficiency by the vector *Aedes albopictus* [9], [10]. Thus, relatively minor consensus genetic changes can significantly influence arbovirus transmission patterns and disease emergence. Determining the mechanistic underpinnings of genetic change in arboviruses is therefore critical to understanding their persistence and emergence.

RNA viruses exist within hosts as a dynamic distribution of non-identical, but related viral variants [11]–[14]. High genetic diversity profoundly influences the population biology of RNA viruses, including WNV, polio, mumps and hepatitis C viruses [15]–[18]. In the case of WNV, high genetic diversity is associated with increased fitness in mosquitoes [19]. Population bottlenecks may reduce fitness by stochastically reducing the genetic diversity of the virus population. *In vitro* studies of vesicular stomatitis virus, an RNA virus, have demonstrated that repeated bottlenecks can lead to fitness loss through the action of Muller's ratchet [20]. The extent to which mosquitoes impose such population bottlenecks on arthropod-borne viruses (arboviruses) is unclear. Analysis of WNV populations from naturally infected birds revealed that non-consensus, minority genotypes were shared among samples collected from multiple birds, suggesting that WNV populations may not be subject to bottlenecks during the natural transmission cycle [14]. Similarly, it was suggested that dengue virus type 1 (DENV1; *Flaviviridae*, *Flavivirus*) is not subject to widespread population bottlenecks during the natural transmission cycle because putatively defective genomes persist through complementation, requiring frequent coinfection of cells in both mosquitoes and humans [21]. Similarly, a high frequency of coinfection of midgut cells has been reported for Venezuelan equine encephalitis virus (VEEV; *Togaviridae*, *Alphavirus*) in *Aedes taeniorhynchus* [22]. Conversely, studies examining early infection of mosquitoes by WNV and VEEV demonstrated that only a few (∼15) midgut cells are susceptible to arbovirus infection [22], [23]. These findings suggest that anatomical barriers, specifically cells of the midgut, may act as genetic bottlenecks by restricting the population of infecting virions thereby diminishing the genetic diversity of the population. Importantly, these observations are not mutually exclusive as several viral genomes may coinfect a single midgut cell. Importantly, population bottlenecks associated with mosquito transmission have not been assessed from a virus genetics perspective.

Therefore, we determined whether WNV experiences genetic bottlenecks during the EIP in the vector mosquito *Culex pipiens quinquefasciatus*. We hypothesized that WNV experiences genetic bottlenecks during the EIP in mosquitoes, and reasoned that sequential reductions in viral genetic diversity would occur as infection progressed throughout the mosquito. To assess this, WNV genetic diversity was quantified in mosquito midguts, hemolymph, and salivary secretions, compartments that represent three well-characterized infection stages (midgut colonization, dissemination, and transmission). Three mosquitoes at three time points (7, 14, and 21 days post infection (dpi)) were sampled. Our data suggest that stochastic reduction of genetic diversity in mosquitoes is at most a minor component of WNV population biology during horizontal transmission.

## Results

### Mosquito Infection Rates

Mosquito tissues were screened for the presence of WNV RNA by one-step RT-PCR. All freshly fed mosquitoes were positive for WNV RNA representing ‘input’, blood-meal associated virus. The infection rates for midguts at 7, 14, and 21 days post infection (dpi), reflecting viral populations able to overcome the midgut infection barrier, were 88% (21/24), 86% (19/22), and 70% (14/20), respectively. The percentage of mosquitoes positive for WNV RNA in the legs, indicating virus dissemination from the midgut and into surrounding hemolymph, was 58% (14/24), 36% (8/22), and 55% (11/20) at 7, 14, and 21 dpi, respectively. In order for mosquitoes to transmit WNV, the virus must be able to overcome the salivary gland infection and escape barriers. The percentage of mosquitoes with WNV in salivary secretions was 25% (6/24), 14% (3/22), and 35% (7/20) at 7, 14, and 21 dpi, respectively. Three mosquitoes per time point with WNV RNA in midgut, hemolymph and saliva were selected for further analysis and WNV genome equivalents quantified (Text S1). Genome equivalents were highest in midguts and progressively decreased in the hemolymph and saliva. Further, genome equivalents increased with time post infection (21 dpi>14 dpi>7 dpi) (Figure S1). In addition, three mosquitoes, representing the ‘input’ group, were collected immediately post-engorgment. WNV genome equivalents determined for each of these individuals were 3.2, 4.1 and 5.9×10^5^ genome equivalents/ml (Figure S1). The bloodmeal contained 6×10^6^ pfu/ml, assuming 10–100 genomes per infectious particle and an engorgment volume of ∼3 µl, engorged mosquitoes would be expected to contain ∼1.4×10^5 or 6^ genome equivalents. The concentrations for the three individuals in the ‘input’ group are in agreement with these calculations and thus faithfully represent the population of the bloodmeal as a whole.

Some arboviruses may enter mosquito hemolymph directly, bypassing midgut infection via a ‘leaky midgut’ [24], [25]. In order to determine whether this occurred in the WNV-*Cx. quinquefasciatus* system, hemolymph was removed from mosquitoes at 1, 3, 24, and 48 hpi as well as 8 and 16 dpi and tested for WNV by plaque assay (Text S1). Hemolymph collected at 8 and 16 dpi commonly held high titers of WNV. In contrast, hemolymph collected at early timepoints after feeding almost never contained infectious WNV (Figure S2).

### WNV Genetic Diversity

**Figure 1:**
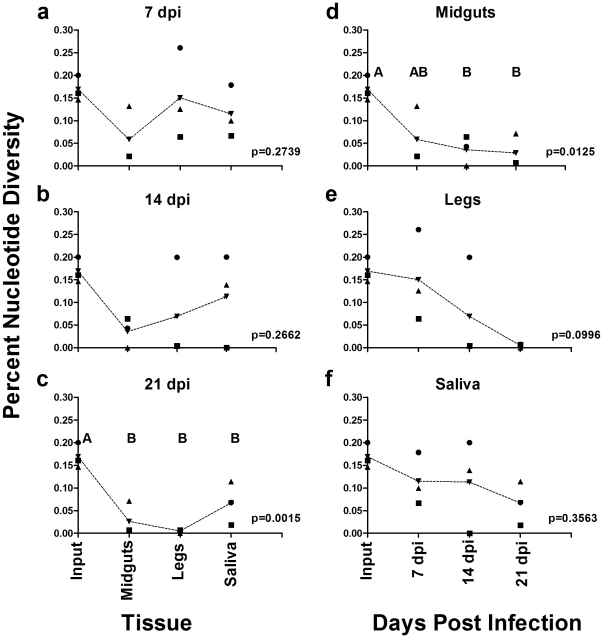
Percent nucleotide diversity by time and tissue. The percent nucleotide diversity was determined for each sample and ploted by either days post infection (7 dpi (A), 14 dpi (B), & 21 dpi (C)) or by tissue type (midguts (D), legs (E), & saliva (F)). Dotted lines connect the means for each sample set. P-values were determined by ANOVA using Tukey's multiple comparison post test. Letters above sample sets represent statistically significant groupings (p-value<0.05). Figures without letters denote that samples were not significantly different from one another.

The percent nucleotide diversity and proportion of unique viral variants were used as indicators of viral genetic diversity in each of the samples. The percent nucleotide diversity was determined by calculating the total number of nucleotide changes for all clones within a given sample divided by the total number of nucleotides sequenced per sample. The data was grouped either by days post infection (Figure 1 A, B, and C) or by tissue type (Figure 1 D, E, and F). Analysis of the data set by days post infection revealed that there was no significant difference in the percent nucleotide diversity among the viral populations sequenced at 7 and 14 dpi between ‘input’, midgut, legs or saliva (p = 0.2739 and p = 0.2662, respectively) (Figures 1 A & B). Interestingly, genetic diversity seemed to decrease with time post infection as there was a significant reduction in diversity from the ‘input’ to the three tissue types at 21 dpi (ANOVA p = 0.0015; Tukey's HSD post test, ‘input’ vs midgut q = 7.262 p<0.05, ‘input’ vs legs q = 8.493 p<0.05, and ‘input’ vs saliva q = 5.293 p<0.05), but no difference between tissue types (Figure 1C). Analyzing the data by tissue type revealed that there was a significant reduction in diversity from the ‘input’ to midguts at 14 and 21 dpi (ANOVA p = 0.0125, Tukey's HSD post test, ‘input’ vs 14 dpi q = 5.404 p<0.05 and ‘input’ vs 21 dpi q = 5.694 p<0.05), but no significant difference between midguts at 7 dpi and ‘input’ (Figure 1D). There was no statistical differences between any of the leg or saliva samples at 7, 14 or 21 dpi (legs p = 0.0996, saliva p = 0.3563) (Figure 1E & 1F).

**Figure 2:**
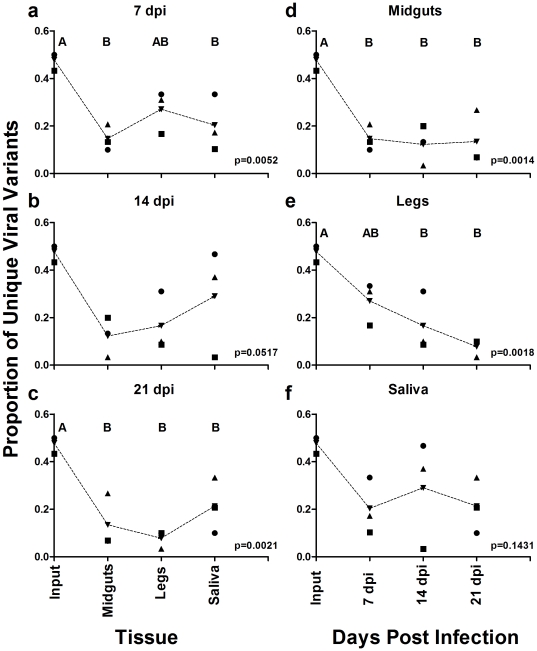
Proportion unique viral variants by time and tissue. The proportion of unique viral variants was determined for each sample and plotted by either days post infection (7 dpi (A), 14 dpi (B), & 21 dpi (C)) or by tissue type (midguts (D), legs (E), & saliva (F)). Dotted lines connect means for each sample set. P-values were determined by ANOVA using Tukey's multiple comparison post test. Letters above sample sets represent statistically significant groupings (p-value<0.05). Figures without letters denote that samples were not significantly different from one another.

The second indicator of genetic diversity used in these studies was the proportion of unique viral variants. This was determined by calculating the number of unique clones per sample and dividing by the total number of clones sequenced per sample. Again the data was grouped either by days post infection (Figure 2 A, B, & C) or tissue type (Figure 2 D, E, & F). At 7 dpi, the midguts and saliva were significantly lower than the ‘input’ (ANOVA p = 0.0052, Tukey's HSD post test, ‘input’ vs midguts q = 7.038 p<0.05, ‘input’ vs saliva q = 5.841 p<0.05), but the tissues were not significantly different from one another (Figure 2A). Interestingly, by 14 dpi the proportion of unique viral variants between the tissues and ‘input’ was not significant (ANOVA p = 0.0517), but at 21 dpi each of three tissue types were significantly lower than the ‘input’ (ANOVA p = 0.0021, Tukey's HSD post test, ‘input’ vs midguts q = 6.898 p<0.05, ‘input’ vs legs q = 8.028 p<0.05, and ‘input’ vs saliva q = 5.306 p<0.05) (Figure 2B & 2C). Analysis by tissue type revealed that midguts from all three time points were significantly lower than the ‘input’, but not different between time points (ANOVA p = 0.0014, Tukey's HSD post test, ‘input’ vs 7 dpi q = 7.298 p<0.05, ‘input’ vs 14 dpi q = 7.838 p<0.05, and ‘input’ vs 21 dpi q = 7.576 p<0.05) (Figure 2D). Like the midguts, the legs at 14 and 21 dpi contained significantly less diversity than the ‘input’ (ANOVA p = 0.0018, Tukey's HSD post test, ‘input’ vs 14 dpi q = 6.597 p<0.05 and ‘input’ vs 21 dpi q = 8.458 p<0.05), but was not different from the 7 dpi time point. Further, there was no difference between the time points (Figure 2E). Finally, comparison of the saliva samples at each of the time points revealed no significant difference between the three time points and the ‘input’ or between time points (ANOVA p = 0.1431) (Figure 2F).

**Figure 3:**
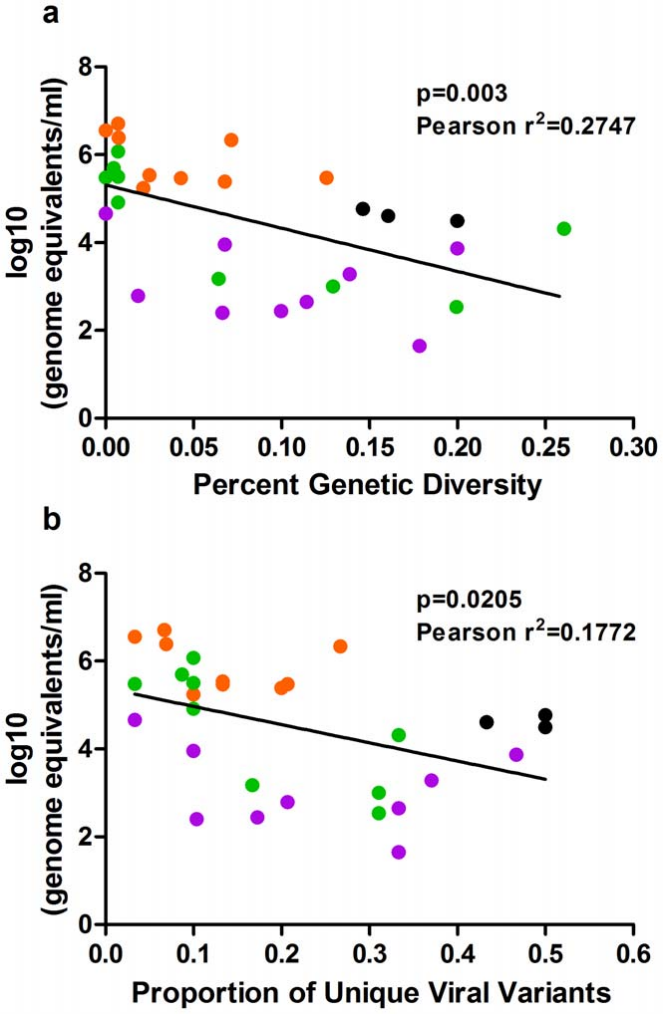
Viral genome equivalents and genetic diversity are inversely correlated. Black = ‘input’, Orange = midguts, Green = legs, and Purple = saliva. (A) Log transformed genome equivalents for each sample plotted against percent nucleotide diversity, n = 30, p = 0.003, Pearson r^2^ = 0.2747. (B) Log transformed genome equivalents for each sample plotted against the proportion of unique viral variants, n = 30, p = 0.0205, Pearson r^2^ = 0.1772.

Because our frequency and location analysis of viral variants revealed that numerous variants were found in both the ‘input’ and saliva, but not the midgut or legs, we performed a correlation analysis between the genetic diversity metrics and log transformed viral genome equivalents. The Pearson correlation analysis revealed that percent nucleotide diversity is significantly inversely correlated to viral genome equivalents (p = 0.003; Pearson r^2^ = −0.2747) (Figure 3A). Similarly, viral genome equivalents are inversely correlated to the proportion of unique viral variants (p = 0.0205; Pearson r^2^ = 0.1772) (Figure 3B).

### Frequency and Migration Analysis

**Figure 4:**
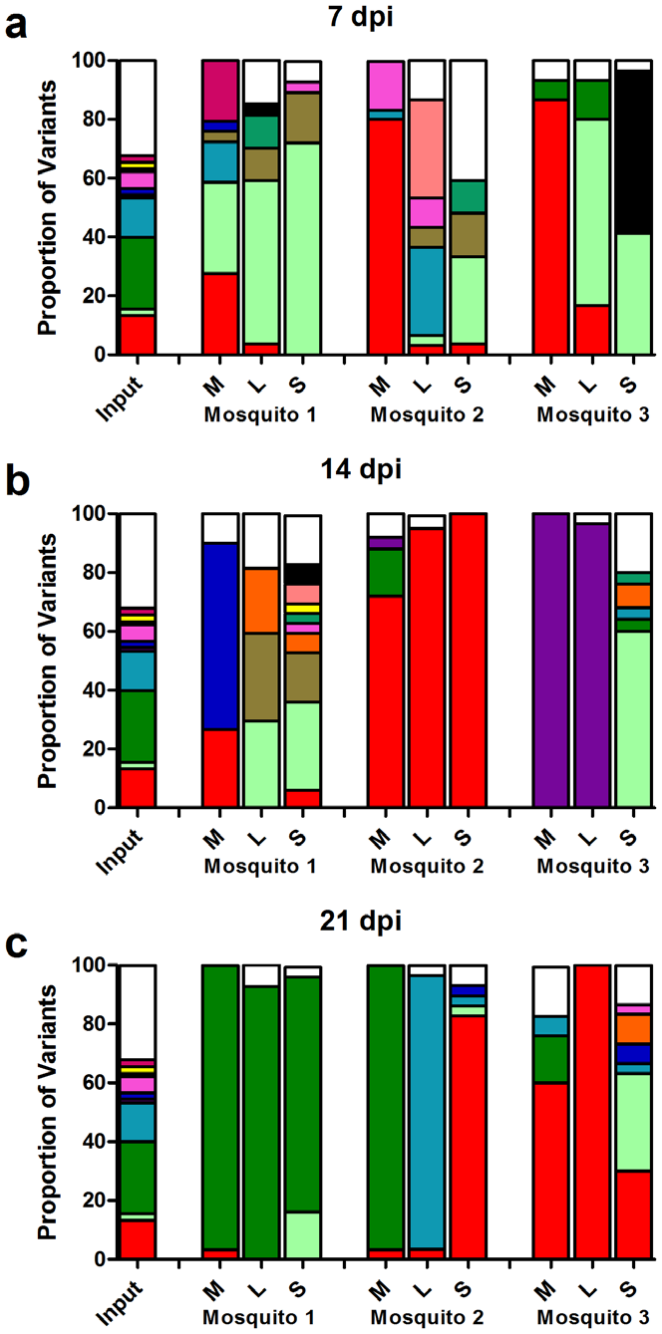
Frequency and location of unique viral variants. There were 883 clones sequenced, of which 78 sequences were unique. The frequency of the fourteen most common viral variants was mapped back to each sample. The column labeled input combines the data from all three 0 hours post infection mosquitoes. Samples are broken down by days post infection (7 dpi (A), 14 dpi (B), & 21 dpi (C)). Each time point includes three mosquitoes (denoted mosquitoes 1–3) and further broken down by tissue (M (midguts), L (legs), & S (saliva)). The white sections of the histograms represent the remaining 64 uncommon variants and the black sections represent a single nucleotide deletion mutant found in multiple samples.

Analysis of the frequency and location of viral variants revealed that 78 of 883 sequences sampled were unique. These variants were found in all three tissue types and at all three time points. There were 16 variants unique to the ‘input’ mosquitoes, 6 were found in all four categories (input, midgut, legs, saliva), 19 were unique to saliva, 15 unique to legs, and 9 unique to midguts, and the remaining variants were found in multiple tissues. The 14 most common variants were then plotted to display their relative proportion in each mosquito sample (Figure 4). By analyzing the data by this approach we were able to track individual variants from ‘input’ through infection (midguts), dissemination (legs), and transmission (saliva). The ‘input’ set is a combination of all three 0 hpi mosquitoes and as expected represents a complex population of multiple variants. Generally, the midgut populations, at all three time points, are composed of only a few variants with no one variant dominating in all samples. Likewise, the WNV populations recovered from the legs had, in general, low intrahost variability, but with no overrepresentation of any one variant between mosquitoes. Interestingly, there was an expansion in the total number of variants identified in the saliva compared to the midguts and legs. These findings are supported by the proportion of unique viral variants analysis (Figure 2). Further, many of the variants that were present in the input samples and subsequently undetected in the midguts and legs were recovered from the saliva (Figure 4). Included in this data was a variant that contained a single nucleotide deletion at nucleotide 2194 in the E-glycoprotein. This deletion mutant was found in the legs or saliva of three different mosquitoes at 7 and 14 dpi (black colored sections of Figures 4A & 4B).

**Figure 5:**
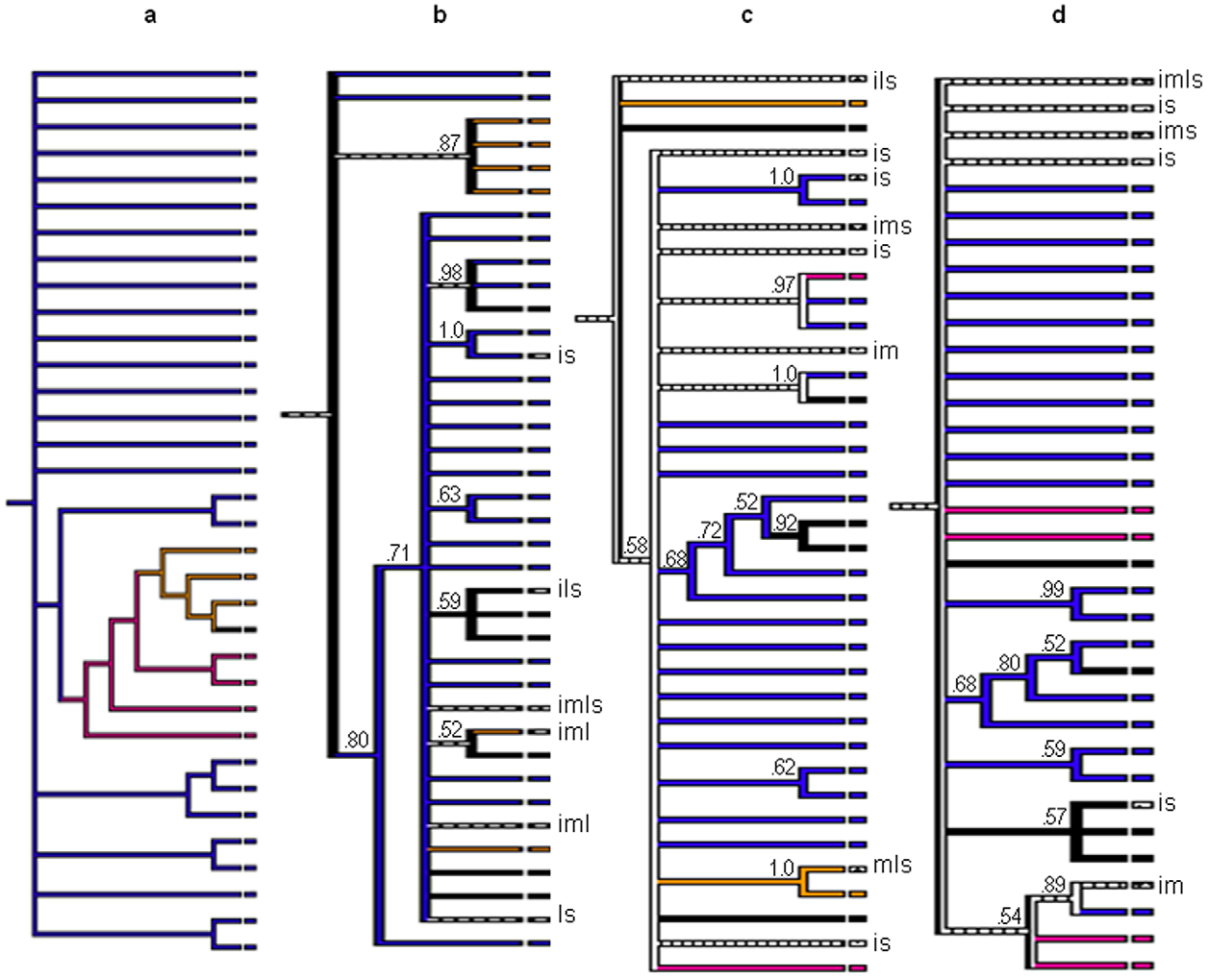
Bayesian trees with the most parsimonious reconstruction of tissue character states for three mosquitoes. Blue = input (i), pink = midgut (m), gold = legs (l), black = salivary secretions (s), dotted = multiple tissues, with specific tissues indicated by abbreviations. Hatched branches indicate equivocal reconstruction of character states. Numbers above nodes are the posterior probabilities inferred for each clade. A) Hypothetical tree with predicted outcome assuming the presence of genetic bottlenecks. The most parsimonious reconstruction of ordered character steps for each tree for three representative mosquitoes is as follows, B) 7 dpi mosquito 2 (22 steps, p<0.0001), C) 14 dpi mosquito 1 (30 steps, p<0.0001), D) 21 dpi mosquito 3 (23 steps, p = 0.0001).

Migration analyses were performed in order to more closely look for evidence of genetic bottlenecks and test for tissue compartmentalization (Figure 5). A hypothetical tree was generated to represent what would be expected if strong genetic bottlenecks were influencing WNV populations during the EIP (Figure 5A). Under this scenario, a single ‘input’ variant initiates infection in the midgut. Subsequently, a single midgut variant establishes an infection in the hemolymph from which a single variant invades the salivary glands and is transmitted. However, this is not what we observed. Three representative mosquitoes, one from each time point, are shown and the remaining six trees are provided in a supplement (Figure S3). The migration analysis indicates some evidence for compartmentalization based on the p-values for ordered character states as estimated from 10,000 randomly sampled trees. A cluster of closely related variants isolated from legs was identified in our representative mosquito at 7 dpi (Figure 5B p<0.0001). Similarly, clusters of saliva variants were identified at 14 dpi and 21 dpi (p<0.0001 and p<0.0001, respectively) (Figures 5C & 5D).

### *In vivo* Competition Assay

To test for the presence of genetic bottlenecks using a less genetically complex virus population, mosquitoes were fed on chicks infected with a known mixture of marked-reference and wild-type WNV (Text S1). The mean proportion of wild-type to reference virus in chick viremia was 0.73 (n = 4, SEM 0.011) (Figure S4). Subsequent analysis of the mosquito samples revealed that there was no change in the proportion of wild-type to reference WNV in any mosquito tissue compared to chick viremia (bodies 0.8 SEM 0.031, legs 0.73 SEM 0.07, and saliva 0.75 SEM 0.087).

## Discussion

Population bottlenecks during transmission may profoundly influence the evolution of arboviruses by stochastically reducing population variation, thereby selecting random genomes that may be less fit than the overall population. Currently, it is unclear whether arboviruses experience genetic bottlenecks during infection of the mosquito vector. Therefore we tracked the composition of WNV populations during mosquito infection to quantify genetic bottlenecks associated with infection of these hosts.

*Cx. p. quinquefasciatus* mosquitoes were exposed to a bloodmeal containing a highly fit, genetically diverse WNV population (M24) that has been described in detail elsewhere [19]. Most mosquitoes exposed to M24 had WNV RNA in midgut tissues after either 7, 14 or 21 days EI. Although fewer mosquitoes had WNV in hemolymph and salivary secretions, at all timepoints at least three individual mosquitoes had WNV in midgut, hemolymph and salivary secretions. WNV from these mosquitoes was used to assess population bottlenecks associated with mosquito transmission.

Extreme care was taken to minimize the possibility that WNV RNA from one tissue would contaminate other tissues. Hemolymph was first sampled from newly anesthetized mosquitoes by gently removing their legs. Second, the mosquito mouthparts were inserted into a pulled capillary tube charged with buffer and the mosquito was allowed to salivate for approximately 30 minutes. Finally, the midgut was removed from the mosquito and washed three times in PBS to remove hemolymph-associated WNV. This approach was validated by intrathoracically inoculating adult female mosquitoes with 2×10^4^ pfu/ml of WNV. Subsequently, five whole mosquitoes, five unwashed midguts and three times washed midguts were collected 45 minutes post inoculation. The presence of WNV RNA was determined by one-step RT-PCR. Expectedly, all five whole body mosquitoes were positive for WNV RNA along with two of five unwashed midguts. All five of the washed midguts were negative for WNV RNA (data not shown). Since the greatest concentration of WNV RNA tended to be in the mosquito midguts, handling this tissue last minimized the possibility of contaminating samples from the same mosquito. Several mosquitoes were detected that had midgut-limited infections, or WNV in hemolymph but not salivary secretions (data not shown). These results indicate that our efforts to minimize contamination were effective and that the samples selected for this study were not compromized by contaminating WNV RNA.

Quantitative analysis of viral genetic diversity in mosquito midguts compared with the highly genetically diverse ‘input’ WNV M24 clearly demonstrated that virus population diversity is restricted in this tissue. Both the percent nucleotide diversity and proportion of unique viral variants in midguts are significantly lower than in the ‘input’ population (Figures 1D & 2D). Surprisingly, however, the genetic diversity of peripheral WNV was not significantly different from virus in the bloodmeal, with saliva-associated WNV (i.e. the WNV that would be transmitted by a feeding mosquito) tending to be the most genetically diverse of the three tissue types sampled (Figures 1 & 2). The mechanism(s) that lead to increased genetic diversity outside of the midgut are not clear. The presence of a ‘leaky midgut’ may explain this discrepency between the percent nucleotide diversity in legs and saliva compared to that of the midguts [24], [25]. However, we found little evidence of WNV bypassing the midgut and directly infecting secondary tissues (Figure S2). In addition, we observed a significant inverse correlation between viral genome equivalents and the genetic diversity metrics (Figure 3). Taken together, these findings suggest that although WNV populations appear to be restricted in the midguts, and to a lesser extent in hemolymph, the genetic diversity of transmitted WNV was similar to that of the ingested virus population, and that variables other than tissue of origin determine viral genetic diversity in mosquitoes.

It may be that WNV accumulates mutations during the course of mosquito infection: relaxation of purifying selection on WNV sequences has been associated with mosquito infection [15]. To assess this possibility, we compared the frequency and location of viral variants present in our mosquito tissue samples to those of the ‘input’ population. Not surprisingly, the majority of the viral variants identified in the midguts were also present in the ‘input’ population (Figure 4). Interestingly, however, variants found in legs and saliva were also represented in our ‘input’ dataset, without being present in the mosquito midguts. These findings support our quantitative analysis of genetic diversity, in these tissues, and indicate that the increased variation observed in peripheral WNV populations was more attributable to genetic diversity in the ‘input’ WNV population than to the generation of novel mutants during mosquito infection.

Additional evidence supporting the infection of a single cell by multiple WNV variants was obtained through examination of a defective WNV sequence in our dataset. Specifically, we identified a single nucleotide deletion mutant that was found in multiple mosquito samples, including peripheral compartments, but not in the ‘input’ (Figure 4). Although it is possible that these mutants arose independently, it seems more likely that an ancestral mutant was present but undetected in the M24 population and was maintained in mosquitoes by complementation. Numerous studies have observed complementation of defective *Flavivirus* genomes in cell passage experiments [26], [27]. Typically, these studies have found large, ∼2 kb, in-frame deletions at the 5′-end of the genome in the structural genes. Interestingly, one study found long-term transmission of a defective DENV-1 virus with a premature stop codon in the E gene [21]. This data suggests that defective WNV particles can infect mosquitoes, propagate through complementation and ultimately be transmitted (Figure 4; mosquito 3 saliva 7 dpi). This implies that multiple WNV virions may frequently infect a single midgut cell, providing a mechanism by which WNV genetic diversity may be maintained in mosquitoes despite limitations in the number of susceptible midgut cells [22], [23].

Finally, we performed a migration analysis to formally test for the presence of bottlenecks and compartmentalization. We detected compartmentalization in legs and saliva, but found no evidence of genetic bottlenecks (Figure 5). In this analysis, if genetic bottlenecks exist, viral variants from the tissue samples would originate from a single ‘input’ variant as demonstrated in our hypothetical tree. Rather, variants identified in the saliva were found to originate from multiple ‘input’ variants indicating the ability of numerous ‘input’ variants to overcome multiple mosquito barriers to infection (i.e. midgut infection, midgut escape, and salivary gland escape barriers). The artificial nature of this experimental system may explain the discrepancies between our tests. Mosquitoes were offered a bloodmeal containing WNV M24 which contains an approximately 10 fold increase in the genetic diversity compared to natural WNV populations [14], [19]. This approach was implemented as a means to more easily track variation in our populations. It may be that the perceived bottlenecks were artificial due to saturating the system. As a more realistic approach to testing for bottlenecks we performed an *in vivo* competition assay in which infectious clone-derived wild-type WNV was competed against a marked reference virus [19]. It was observed that the proportion of marked refernce virus to wild-type WNV remained unchanged from ‘input’ to bodies, legs or saliva (Figure S4). Together, these data suggest that genetic bottlenecks do not significantly influence WNV populations during the EIP in *Cx. p. quinquefasciatus*.

Our genetic approach to transmission bottlenecks provides an intersting contrast to previous studies of bottlenecks in arbovirus transmission cycles [22], [23]. Using virus-like particles to track binding and internalization, one study demonstrated that WNV infects only a few midgut epithelial cells during infection of *Cx. quinquefasciatus* [23]. Similar results were found during VEEV infection of *Aedes taeniorhynchus* [22]. By virtue of the small number of infected cells it was concluded that arbovirus populations may be stochastically reduced at the point of infection. Our genetics studies of WNV do not support this obsevation. Notably, these conclusions are not necessarily mutually exclusive: It may be that the small proportion of susceptible midgut cells are infected with more than one virus particle or that an undetectable level of infection occurred in a higher proportion of cells. In fact, a high frequency of dual infections were observed in the VEEV-*Aedes* system [22]. Essentially, only a few susceptible midgut cells may be needed to propagate a diverse arbovirus population. Our observation of a deletion mutant persisting, apparently through complementation, during mosquito infection supports this possibility.

The literature regarding the role of bottlenecks in natural transmission cycles of RNA viruses is currently ambiguous. Bottlenecks are seemingly unimportant for Cauliflower mosaic virus in plants, but may exist for other RNA viruses [28]–[31]. Numerous factors may contribute to this discrepency such as virus species, single vs two-host systems, mode of transmission and/or site of inoculation. Further, environmental or host genetic factors may influence differences between individual hosts within a given population and likely explain the high variablity observed between individual mosquitoes in this experiment [32]. Nevertheless, our data establish that transmitted WNV populations are at least as diverse as those of the imbibed population and therefore suggests that genetic bottlenecks are unlikely to significantly influence WNV population biology during horizontal transmission.

## Materials and Methods

### Virus and Mosquito Infections

The highly genetically diverse WNV population, WNV M24, used for these studies has been previously described [19]. Briefly, 24 WNV isolates from naturally infected mosquitoes and birds were passaged once on C6/36 *Aedes albopictus* cells [14]. Titers were determined by plaque assay on Vero cells and mixed at a 1∶1∶1… ratio. This mixture was amplified once on C6/36 cells at an MOI of 0.1 and the resultant population titered and genetically characterized.

To infect mosquitoes, WNV M24 was mixed 1∶1 with defibrinated goose blood and offered to adult female *Culex pipiens quinquefasciatus* 7–8 days post emergence. The virus titer in the bloodmeal was 6×10^6^ pfu/ml. Fully engorged females were separated from the remaining unfed mosquitoes and housed in an environmental chamber (27°C, 16∶8 L∶D photoperiod) for the remainder of the experiment.

### Sample Collection and Virus Detection

To quantify viral genetic diversity of the ‘input’ virus population, three fully engorged mosquitoes were placed in RNA extraction buffer immediately after feeding and homogenized using a mixer mill. Viral genetic diversity was quantified as described below. At 7, 14, and 21 days post-infection paired tissues samples (midguts, legs, and saliva) were collected from 20–25 mosquitoes. To ensure that contaminating WNV from the hemoceol was not introduced into our midgut samples, dissected midguts were washed three times in PBS before placing the samples in RNA extraction buffer. Dissecting forceps were flame sterilized between dissections to avoid cross contamination between samples. Total RNA was extracted from mosquito hemolymph and tissues using the RNeasy Mini Protect kit (Qiagen, Valencia, CA) and screened for the presence of WNV RNA by one-step RT-PCR using the Superscript III kit with platinum *Taq* (Invitrogen, Carlsbad, CA). WNV specific primers used in this study spanned a 934 nt. region corresponding to the E-NS1 junction (1971 nt–2928 nt). Three mosquitoes with detectable WNV RNA in all three tissue types were selected from each time point for further analyses.

### Quantification of Viral Genetic Diversity

Viral genetic diversity was determined according to methods previously described [14]. Briefly, cDNA was generated from 5 µl of total RNA using the High Fidelity Reverse Transcription kit (Stratagene, Cedar Creek, TX) according to the manufacturers specifications and WNV specific primers, WNV 1971 F and WNV 2928 R. Subsequently, the cDNA served as template for high fidelity *Pfu* Ultra polymerase amplification (Stratagene). Amplicons were PCR purified and cloned into the pCR Script Amp ^(+)^ vector (Stratagene). Between 21–30 individual clones from each of the samples were sequenced using the M13F, M13R, WNV 2369 F, and WNV 2768 R primers. DNAStar's SeqMan module (DNAStar Inc., Madison, WI) was used for sequence alignment and analysis of genetic diversity. Only clones with two-fold sequencing coverage were considered complete. As a means to estimate genetic diversity, consensus sequences for each sample were determined and individual clones within that sample were then compared to the specimen-specific consensus sequence. The percent nucleotide diversity (total number of mutations from all clones within a sample divided by the total number of nulcoetides sequenced per sample) and the proportion of unique viral variants (the number of unique clones differing from the consensus divided by the total number of clones sequenced per sample) were calculated and used as indicators of genetic diversity.

### Quantification of Viral Genome Equivalents

WNV genome equivalents were determined by quantitative-RT-PCR (Q-RT-PCR). As a standard control for this assay a ∼2 kb fragment from the WNV E gene was amplified using the WNV 1031 F and WNV3430 R primers. The resultant amplicon was cloned into the pCR2.1-TOPO vector (Invitrogen) downstream of the T7 promoter. The recombinant vector was linearized with *Kpn* I, purified and used as template for *in vitro* transcription using the T7 Megascript kit according the manufacturer's instructions (Ambion, Austin, TX). The resultant RNA was quantified and aliquoted in serial ten-fold dilutions. Using a probe specific for the E gene, the WNV 1160 F and WNV 1229 R primers, and the TaqMan ® One-Step RT-PCR Master Mix Reagent (Applied Biosystems, Foster City, CA) viral RNA copy numbers were determined [33]. Samples were run on the ABI Prism 7000 Sequence Detection System (Applied Biosystems).

### Frequency and Migration Analysis

The presence of genetic bottlenecks and/or compartmentalization was further assessed by migration analyses. To determine the frequency and location of viral variants, sequences from each sample were aligned in DNAStar's SeqMan module, exported as FASTA files and duplicates removed using BioEdit [34]. Alignments were generated for each mosquito and tested for recombination using the Genetic Algorithm for Recombination detection program implemented on the datamonkey.org website [35]. Evidence of recombination was not detected, so the alignments were used to perform a migration analysis. To test the null hypothesis of panmixis versus the alternative that there are distinct WNV sub-populations within different mosquito tissues, we used the Slatkin-Maddison test for gene flow in MacClade version 4 (Sinauer Associates, Sunderland, MA) [36]. Tissue of origin was assigned to each taxon in a one-character data matrix. ‘Input’ sequences from freshly-fed mosquitoes were included as an estimate of the population of variants present in the infectious bloodmeal. In total there were four character states (input, midgut, legs, and saliva). The Slatkin-Maddison test was performed independently for each mosquito. This analysis was performed on Bayesian phylogenies, generated with MrBayes 3.1.2 [37]. These were run with a general time reversible (GTR) model with invariable rates with substitution rates following a gamma plus invariants distribution. Two Markov Chains Monte Carlo (MCMC) tree searches of 5 million generations each were run in parallel with sampling one in every 250 trees. 50% majority-rule consensus trees are shown based on the last 19,000 trees. Briefly, the phylogenetic tree resulting from the nucleotide data was loaded into MacClade and the most parsimonious reconstruction of this ancestral character inferred with the Fitch algorithm [38] in order to estimate the minimum number of steps required to explain the distribution of tissue states on the tree of interest. We then generated 10,000 random trees by random joining and splitting of the input tree and compared the number of steps on our input tree to those calculated in the random trees, as described previously for HIV-1, using ordered tissue states [39].

### Statistical Analysis

Statistical analyses were completed in Microsoft Excel and GraphPad Prism. A one-way analysis of variance (ANOVA) with the Tukey's multiple comparison post-test with a significance level of α = 0.05 was used for analysis of the percent nucleotide diversity and proportion of unique viral variants. A Pearson correlation analyses was perfomed on log transformed viral genome equivalents versus percent nucleotide diversity and proportion of unique viral variants. Figures were generated in GraphPad.

## Supporting Information

Figure S1 **WNV genome equivalents per tissue sample.** WNV genome equivalents were determined by Q-RT-PCR for each sample characterized.
(TIF)

Figure S2 **WNV titers in** ***Culex pipiens quinquefasciatus*** **hemolymph at early time points.** Mosquitoes were offered a WNV Mix24 infectious bloodmeal and hemolymph extracted at multiple time points. WNV titers were determined by plaque assay.
(TIF)

Figure S3 **Bayesian trees with the most parsimonious reconstruction of tissue character states from the six remaining mosquitoes.** Blue = input (i), pink = midgut (m), gold = legs (l), black = salivary secretions (s), dotted = multiple tissues, with specific tissues indicated by abbreviations. Hatched branches indicate equivocal reconstruction of character states. Numbers above the nodes are the posterior probabilities inferred for each clade. Mosquito analyzed and most parsimonious reconstruction of ordered character steps for each tree is as follows A) 7 dpi mosquito 1 (21 steps, p = 0.0002) B) 7 dpi mosquito 3 (10 steps, p = 0.0057), C)14 dpi mosquito 2 (8 steps, p = 0.087), D) 14 dpi mosquito 3 (23 steps, p = 0.0002), E) 21 dpi mosquito 1 (12 steps, p = 0.0772), F) 21 dpi mosquito 2 (16 steps, p = 0.007).
(TIF)

Figure S4 **The proportion of wild-type WNV when competed against a marked reference virus does not change as the virus disseminates through the mosquito.** *Culex pipiens quinquefasciatus* mosquitoes were fed on live chicks circulating a mixed population of WNV comprised of wild-type (WT) and reference viruses. Tissues were harvested 7 dpi from 20 mosquitoes and the proportion of WT-WNV was determined by RT-PCR followed by SNPS analysis. Samples negative for WNV RNA by RT-PCR were omitted.
(TIF)

Text S1 **Materials and Methods.**
(DOC)
