# Supplementary figures and images for: West Nile Virus Genetic Diversity is Maintained during Transmission by Culex pipiens quinquefasciatus Mosquitoes
Source: PLoS One. 2011 Sep 12;6(9):e24466. doi: 10.1371/journal.pone.0024466 (PMC3171416; doi:10.1371/journal.pone.0024466)

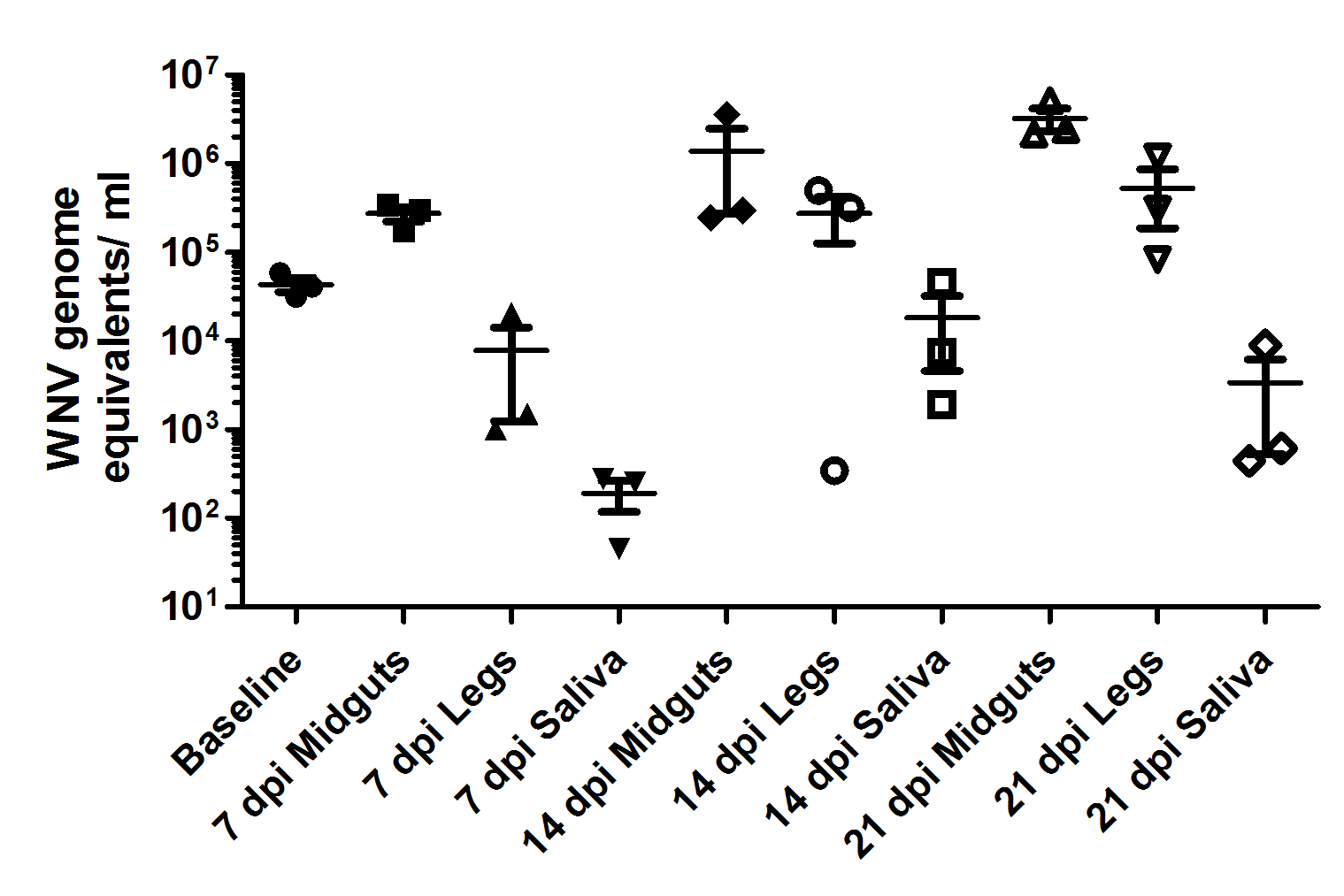

Supplement: Figure S1 — WNV genome equivalents per tissue sample. WNV genome equivalents were determined by Q-RT-PCR for each sample characterized. (TIF) [file pone.0024466.s001.tif]

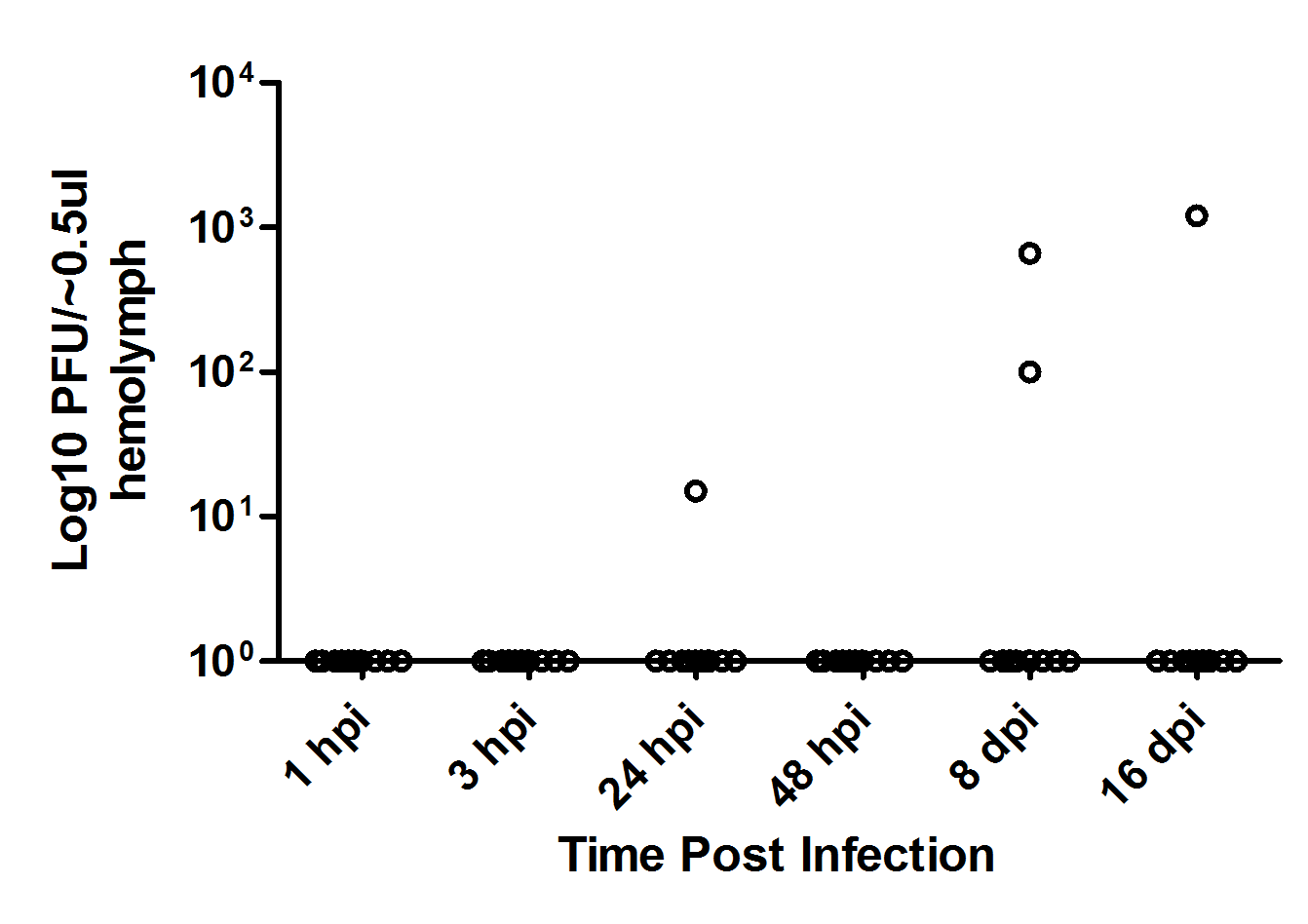

Supplement: Figure S2 — WNV titers in Culex pipiens quinquefasciatus hemolymph at early time points. Mosquitoes were offered a WNV Mix24 infectious bloodmeal and hemolymph extracted at multiple time points. WNV titers were determined by plaque assay. (TIF) [file pone.0024466.s002.tif]

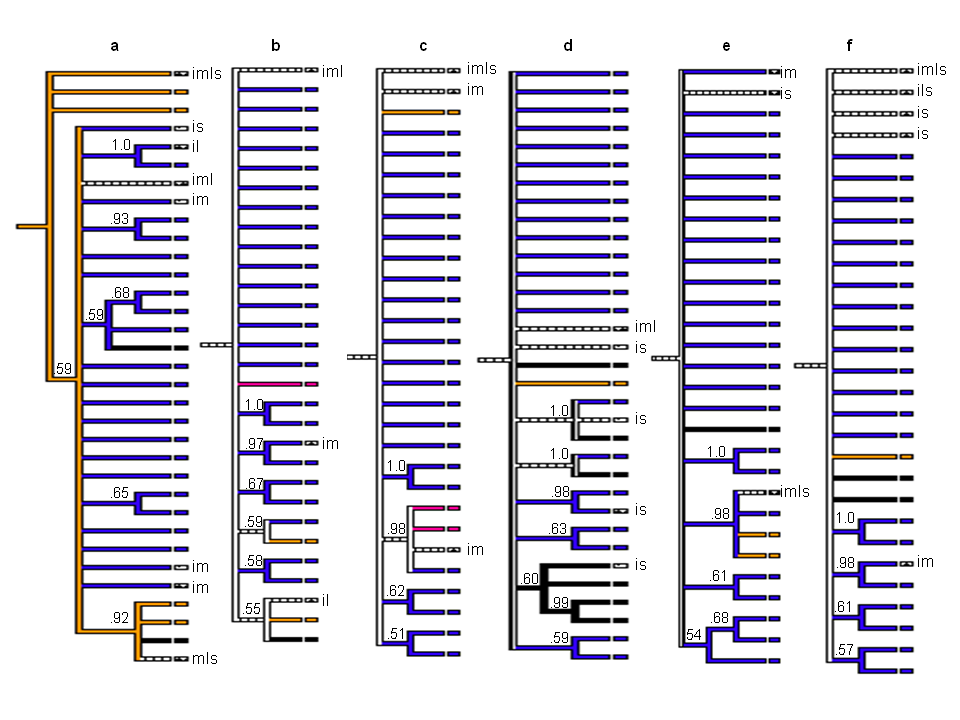

Supplement: Figure S3 — Bayesian trees with the most parsimonious reconstruction of tissue character states from the six remaining mosquitoes. Blue = input (i), pink = midgut (m), gold = legs (l), black = salivary secretions (s), dotted = multiple tissues, with specific tissues indicated by abbreviations. Hatched branches indicate equivocal reconstruction of character states. Numbers above the nodes are the posterior probabilities inferred for each clade. Mosquito analyzed and most parsimonious reconstruction of ordered character steps for each tree is as follows A) 7 dpi mosquito 1 (21 steps, p = 0.0002) B) 7 dpi mosquito 3 (10 steps, p = 0.0057), C)14 dpi mosquito 2 (8 steps, p = 0.087), D) 14 dpi mosquito 3 (23 steps, p = 0.0002), E) 21 dpi mosquito 1 (12 steps, p = 0.0772), F) 21 dpi mosquito 2 (16 steps, p = 0.007). (TIF) [file pone.0024466.s003.tif]

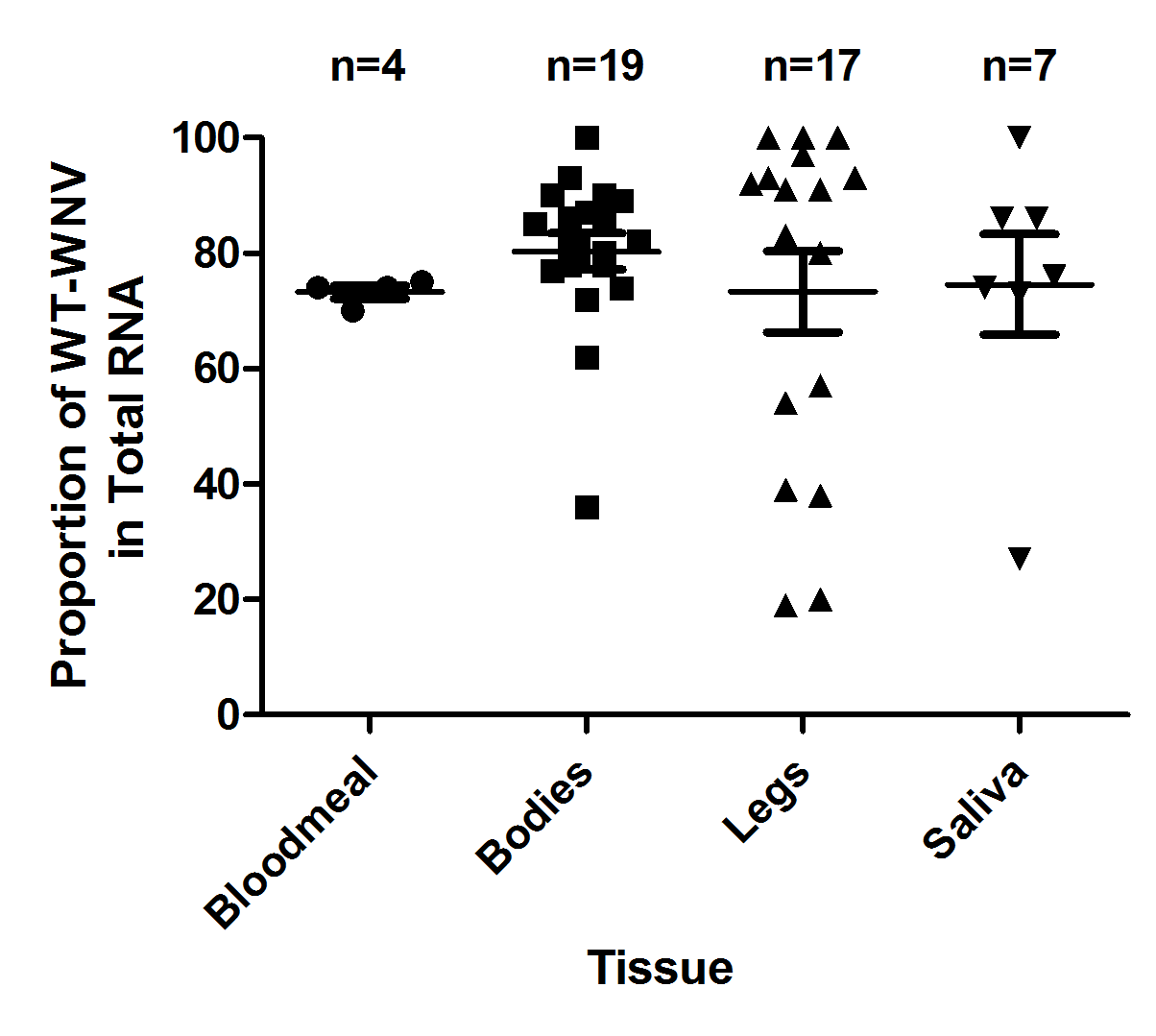

Supplement: Figure S4 — The proportion of wild-type WNV when competed against a marked reference virus does not change as the virus disseminates through the mosquito. Culex pipiens quinquefasciatus mosquitoes were fed on live chicks circulating a mixed population of WNV comprised of wild-type (WT) and reference viruses. Tissues were harvested 7 dpi from 20 mosquitoes and the proportion of WT-WNV was determined by RT-PCR followed by SNPS analysis. Samples negative for WNV RNA by RT-PCR were omitted. (TIF) [file pone.0024466.s004.tif]
