# Supplementary material for: West Nile Virus Genetic Diversity is Maintained during Transmission by Culex pipiens quinquefasciatus Mosquitoes
Source: PLoS One. 2011 Sep 12;6(9):e24466. doi: 10.1371/journal.pone.0024466 (PMC3171416; doi:10.1371/journal.pone.0024466)
Supplement: Text S1 — Materials and Methods. (DOC) [file pone.0024466.s005.doc]

Quantification of Viral Genome Equivalents

WNV genome equivalents were determined by quantitative-RT-PCR (Q-RT-PCR). As a standard control for this assay a ~2 kb fragment from the WNV E gene was amplified using the WNV 1031 F and WNV3430 R primers. The resultant amplicon was cloned into the pCR2.1-TOPO vector (Invitrogen) downstream of the T7 promoter. The recombinant vector was linearized with *Kpn* I, purified and used as template for *in vitro* transcription using the T7 Megascript kit according the manufacturer's instructions (Ambion, Austin, TX). The resultant RNA was quantified and aliquoted in serial ten-fold dilutions. Using a probe specific for the E gene, the WNV 1160 F and WNV 1229 R primers, and the TaqMan ® One-Step RT-PCR Master Mix Reagent (Applied Biosystems, Foster City, CA) viral RNA copy numbers were determine ^1^. Samples were run on the ABI Prism 7000 Sequence Detection System (Applied Biosystems).

Hemolymph Collection and Titration

Colonized *Cx. p. quinquefasciatus* mosquitoes were reared according to standard laboratory procedures. Females that had been starved for ~36h were allowed to feed on infectious clone-derived WNV mixed 1:1 with defibrinated goose blood (Rockland Immunochemicals Inc., Gilbertsville, PA). Engorged mosquitoes were separated and held for 1, 3, 24 or 48 hours; or 8 or 16 days extrinsic incubation. Hemolymph was sampled from mosquitoes anesthetized with triethylamine (Sigma) by inserting a glass needle into the posterior portion of the thorax, adjacent to the abdomen. Care was taken to avoid rupturing the midgut, and coloration of the hemolymph that might indicate such was noted. Approximately 0.3-0.5ul of hemolymph was sampled per mosquito. Hemolymph was expelled from the needle into 100ul of mosquito diluent and stored at -80 until use. Infectious virus in hemolymph samples was quantified by plaque assay on Vero cells according to standard methods.

*In vivo* Competition Assays

Equal titers of an infectious clone-derived marked reference virus and infectious clone derived wild-type virus were mixed and ~2000 PFU subcutaneously inoculated into chicks at a concentration ^2,3^. Two days post infection female *Culex pipiens quinquefasciatus* were fed on the infected chicks. At 7 dpi mosquito bodies, legs and saliva were collected, RNA extracted, and the proportion of WT-WNV determined by RT-PCR followed by SNPS analysis ^4^.

**REFERENCES**

1. Lanciotti, R.S. et al. Rapid detection of West Nile virus from human clinical specimens, field-collected mosquitoes, and avian samples by a TaqMan reverse transcriptase-PCR assay. *Journal of Clinical Microbiology* **38**, 4066-4071 (2000).

2. Fitzpatrick, K.A. et al. Population variation of West Nile virus confers a host-specific fitness benefit in mosquitoes. *Virology* **404**, 89-95 (2010).

3. Shi, P.Y., Tilgner, M., Lo, M.K., Kent, K.A. & Bernard, K.A. Infectious cDNA clone of the epidemic West Nile virus from New York City. *Journal of Virology* **76**, 5847-5856 (2002).

4. Hall, G.S. & Little, D.P. Relative quantitation of virus population size in mixed genotype infections using sequencing chromatograms. *Journal of Virological Methods* **146**, 22-28 (2007).
